# Data on modeling mycelium growth in Pleurotus sp. cultivation by using agricultural wastes via two level factorial analysis

**DOI:** 10.1016/j.dib.2018.09.008

**Published:** 2018-09-08

**Authors:** Noor Athirah Dzulkefli, Norazwina Zainol

**Affiliations:** Faculty of Chemical & Natural Resources Engineering, Universiti Malaysia Pahang, 26300 Gambang, Pahang, Malaysia

## Abstract

In this article, five variables including type of substrates, sizes of substrates, mass ratio of spawn to substrates (SP/SS), temperature and pretreatment of substrates were used to model mycelium growth in Pleurotus sp. (oyster mushroom) cultivation by using agricultural wastes via two level factorial analysis. Two different substrates which were empty fruit bunch (EFB) and sugarcane bagasse (SB) were used. Analysis of Variance (ANOVA) for both mycelium extension rate (*M*) and nitrogen concentration in mycelium (*N*) showed that the confidence level was greater than 95% while *p*-value of both models were less than 0.05 which is significant. The coefficient of determination (*R*^2^) for both *M* and *N* were 0.8829 and 0.9819 respectively. From the experiment, the best condition to achieve maximum *M* (0.8 cm/day) and *N* (656 mg/L) was by using substrate B, 2.5 cm size of substrate, 1:14 for SP/SS, incubated at ambient temperature and application of steam treatment. The data showed that EFB can be used to replace sawdust as a media for the oyster mushroom cultivation. Data analysis was performed using Design Expert version 7.0.

**Specifications table**

**Table:** 

*Subject area*	*Environmental Science*
*More specific subject area*	*Biotechnology*
*Type of data*	*Figure and Table*
*How data was acquired*	*The substrates were cut (1.5 cm and 2.5 cm), then soaked in water overnight, filtered to drain excess water and weighed at 100 g. Then, the substrates were pretreated (application of steam or no treatment) and inoculated with spawn by placing the spawn on the surface of substrate according to the selected mass ratio of spawn to substrates. The bottles were closed and incubated (at ambient or 25* °*C) in the dark condition. Then, mycelium extension was measured and HACH spectrophotometer was used to analyze nitrogen concentration in the substrate. Experimental data were analyzed by using Design Expert software in order to determine the most contributing factors.*
*Data format*	*Raw, analyzed*
*Experimental factors*	*The main and interaction effects of type of substrate, sizes of substrates, mass ratio of spawn to substrates (SP/SS), temperature and pretreatment of substrates to mycelium extension rate (M) and nitrogen concentration in mycelium (N) were evaluated.*
*Experimental features*	*Mycelium growth extension rate was determined and nitrogen concentration in mycelium was analyzed*
*Data source location*	*Universiti Malaysia Pahang*
*Data accessibility*	*All data are available within the paper*

**Value of the data**

• Agricultural wastes were used for mycelium growth of Pleurotus sp. cultivation.
• The data will be useful for application of agricultural wastes in industrial Pleurotus sp. cultivation. Agricultural wastes such as empty palm fruit bunch (EFB) and sugarcane bagasse (SB) are among the most abundant wastes in Malaysia. Cultivation of Pleurotus sp. (oyster mushroom) by using agricultural wastes is one of the sustainable methods with low cost substrate.
• This data will be useful to researchers and scientific community wanting to analyze the ability of EFB in Pleurotus sp. cultivation.

## 1 Data

The obtained data in this paper indicated the mycelium growth of Pleurotus sp. cultivation by using agricultural wastes. Five studied variables (type of substrates, sizes of substrate, mass ratio of spawn to substrate (SP/SS), temperature and pretreatment of substrates) and their levels have been shown in Table 1. Meanwhile, Table 2 shows the experimental design by Design Expert software version 7.0 and data of both responses of mycelium extension rate (*M*) and nitrogen concentration (*N*). From the experiment, the best condition obtained for maximum *M* (0.8 cm/day) and *N* (656 mg/L) was by using substrate B, 2.5 cm size of substrate, 1:14 for SP/SS, incubated at ambient temperature and application of steam treatment. According to the percentage contribution factor for *M* and *N*, pretreatment of substrate (59.30%) and type of substrate (75.80%) have the highest contribution, respectively. This is supported by the *p*-value of main effects of all five studied variables that obtained by Analysis of Variance (ANOVA), pretreatment of substrates on *M* and type of substrate on *N* was statistically significant (*p*-value < 0.05). The effects of studied variables on *M* and *N* have been shown in Figs. (2) and (7). The equations for both *M* and *N* by using type of substrates (A), sizes of substrate (B), SP/SS (C), temperature (D) and pretreatment of substrates (E) are as Eqs. (1), (2) respectively;

(1) $$\begin{array}{ccc} M\;\left(cm/day\right) & \;= & \;0.60\;-\;0.023A\;+\;0.019B\;-\;0.029C\;+\;0.029D\;-\;0.14E\\ & & \;+\;0.041BD\;-\;0.036BE\;-\;0.044CE\;+\;0.044DE \end{array}$$

(2) N(mg/L)=352+241.5A+43B−0.50C+50.50D+21.50E+59.50AB+41.00AD−35.50BC+47.50BE−33.00CD−39.00CE+32.00DE

**Table 1:** Five studied variables and levels.

**Independent variables**	**Unit**	**Factors**	**Low level**	**High level**
Type of substrate	–	A	A (SB)	B (EFB)
Size of substrate	cm	B	0.5	2.5
Mass ratio of spawn to substrate (SP/SS)	–	C	1:10	1:14
Temperature	°C	D	25	Ambient (28–30)
Pretreatment of substrate	–	E	Steam	Non-steam

**Table 2:** Experimental design by using TLFA by Design Expert software (Version 7) and responses values.

*Run*	*Factor 1*	*Factor 2*	*Factor 3*	*Factor 4*	*Factor 5*	*Response 1*				*Response 2*			
	A: Type of substrate	B: Size of substrates (cm)	C: SP/SS (g:g)	D: Temperature	E: Pre-treatment of substrate	Mycelium extension rate (*M*) (cm/day)				Nitrogen concentration (*N*) (mg/L)			
						1	2	3	Avg	1	2	3	Avg
5	A	0.5	1:10	25 °C	Steam	0.78	0.78	0.78	0.78	136	120	132	128
8	B	0.5	1:10	25 °C	Non-Steam	0.67	0.62	0.47	0.58	344	360	368	356
9	A	2.5	1:10	25 °C	Non-Steam	0.35	0.33	0.28	0.32	120	124	116	120
14	B	2.5	1:10	25 °C	Steam	0.80	0.80	0.80	0.80	440	444	532	472
3	A	0.5	1:14	25 °C	Non-Steam	0.38	0.38	0.35	0.37	88	80	72	80
13	B	0.5	1:14	25 °C	Steam	0.69	0.66	0.57	0.64	580	568	564	572
4	A	2.5	1:14	25 °C	Steam	0.80	0.80	0.80	0.80	76	80	72	76
2	B	2.5	1:14	25 °C	Non-Steam	0.36	0.21	0.27	0.28	604	616	600	608
15	A	0.5	1:10	Ambient	Non-Steam	0.60	0.60	0.60	0.60	132	144	132	136
7	B	0.5	1:10	Ambient	Steam	0.43	0.58	0.58	0.53	476	476	476	476
16	A	2.5	1:10	Ambient	Steam	0.79	0.79	0.79	0.79	76	92	108	92
11	B	2.5	1:10	Ambient	Non-Steam	0.62	0.65	0.65	0.64	1044	1052	1028	1040
6	A	0.5	1:14	Ambient	Steam	0.80	0.77	0.88	0.79	160	172	160	164
12	B	0.5	1:14	Ambient	Non-Steam	0.36	0.36	0.36	0.36	552	568	556	560
1	A	2.5	1:14	Ambient	Non-Steam	0.56	0.53	0.53	0.54	88	88	88	88
10	B	2.5	1:14	Ambient	Steam	0.79	0.80	0.78	0.79	648	664	676	664

1:10 SP/SS (10 g spawn for 100 g substrate).

1:14 SP/SS (7 g spawn for 100 g substrate).

## 2 Experimental design, materials and methods

### 2.1 Collection of substrates and spawns

Five bunches empty palm fruit bunch (EFB) were collected from palm oil plantation at Banting, Selangor. Meanwhile, 800 g of sugarcane bagasse (SB) was collected at Semenyih, Selangor. 400 g of Pleurotus sp. spawn has been purchased from mushroom grower at Kuantan, Pahang.

### 2.2 Experimental design for factorial analysis

There were five selected factors that give contribution to oyster mushroom growth (Table 1). The factors were type of substrates, size of substrates, mass ratio of spawn to substrates (SP/SS), temperature and pre-treatment of substrates. The experimental design in Table 2 was constructed by using Design Expert software where all the factors were randomized [2]. SB (A) and EFB (B) were prepared in bottles according to run in Table 2. Firstly, the substrates were cut into the selected size, then soaked in water for overnight, filtered to drain excess water and weighed for 100 g. Then, the substrates were pretreated with selected treatment and inoculated with spawn by placing the spawn on the surface of substrate. The bottles were closed and incubated at selected temperature in dark condition. The experiment was conducted according to set-up in Table 2. There were two responses which were mycelium extension rate (*M*) and nitrogen concentration in mycelium (*N*). Experimental data were analyzed using the same software in order to determine the most contributing factors.

### 2.3 Sample analysis

Sample analysis was conducted after the bottle fully colonized with mycelium. There were two responses which were mycelium extension rate (*M*) and nitrogen concentration in mycelium (*N*).

#### 2.3.1 Determination of mycelium growth

Mycelium growth was measured in centimeters as the length of the mycelium spreading from the surface of substrates toward the bottom of bottles [1]. The equation for mycelium extension rate (*M*) is as Eq. (3);

(3) $$\mathrm{Mycelium\backslash extension\backslash rate}\;(M)\;(\frac{\mathrm{cm}}{\mathrm{day}})\;=\;\frac{\mathrm{Length\backslash of\backslash mycelium}}{\mathrm{Days\backslash of\backslash complete\backslash mycelium}\;\mathrm{growth}}$$

#### 2.3.2 Nitrogen concentration analysis by using HACH Spectrophotometer

*N* was determined by using Persulfate Digestion Method (Method 10072). The mycelium was collected from the substrates and diluted by using deionized water. Then, *N* was analyzed by using HACH Spectrophotometer.

### 2.4 Data analysis

All data obtained were recorded in Design Expert software. The responses were analyzed using ANOVA based on *p*-value with 95% of confidence level to identify the most contributing factors and interaction between the factors that affect both responses *M* and *N.*

#### 2.4.1 Mycelium extension rate (*M*)

**Fig. 1:**
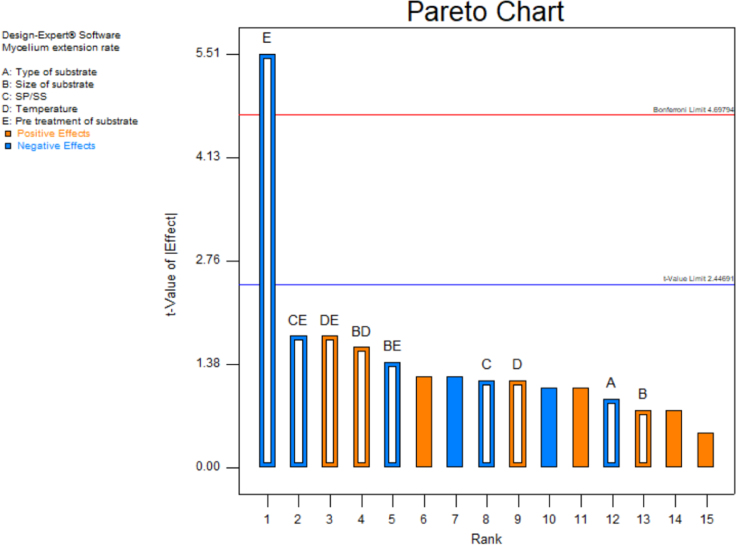
Pareto chart of mycelium growth extension rate (*M*).

**Fig. 2:**
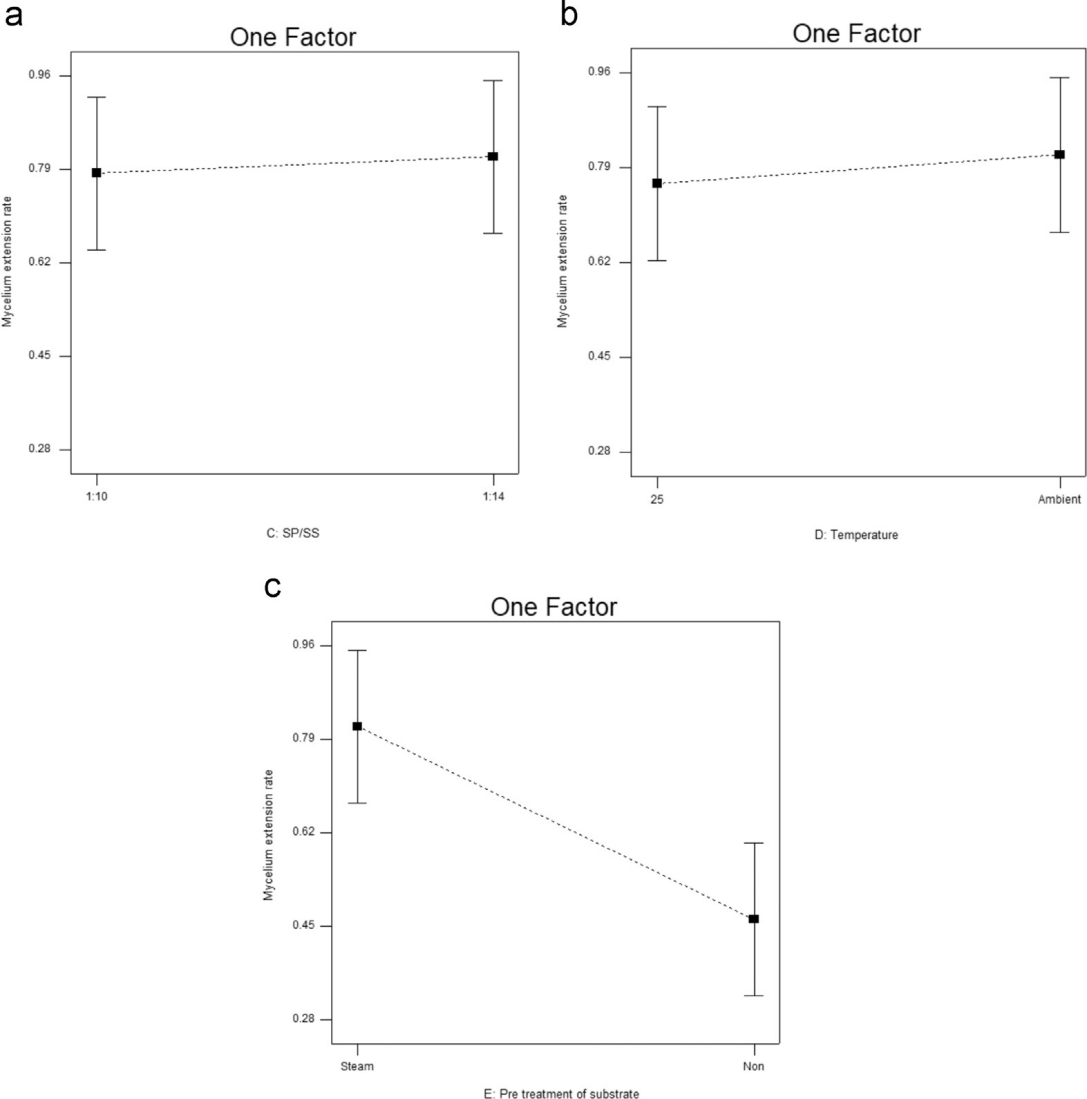
Effect of most contributing factors to mycelium extension rate (*M*).

**Fig. 3:**
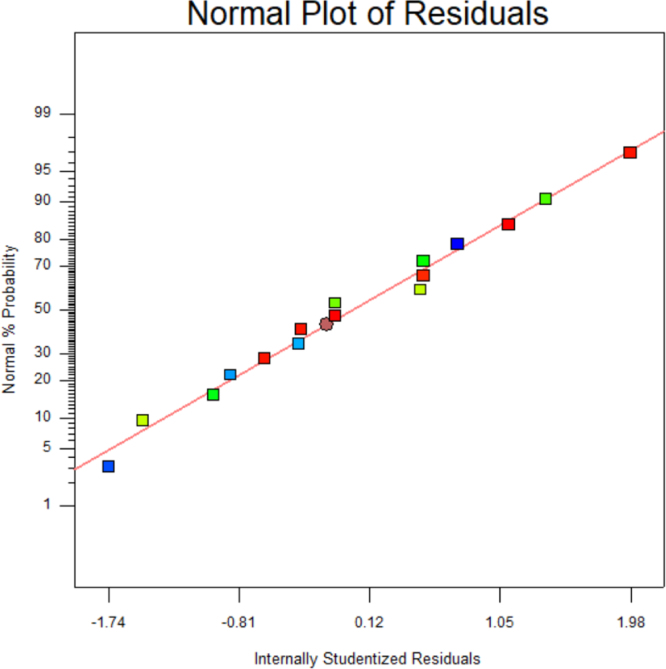
Normal probability plots of internally studentized residuals for mycelium extension rate (*M*).

**Fig. 4:**
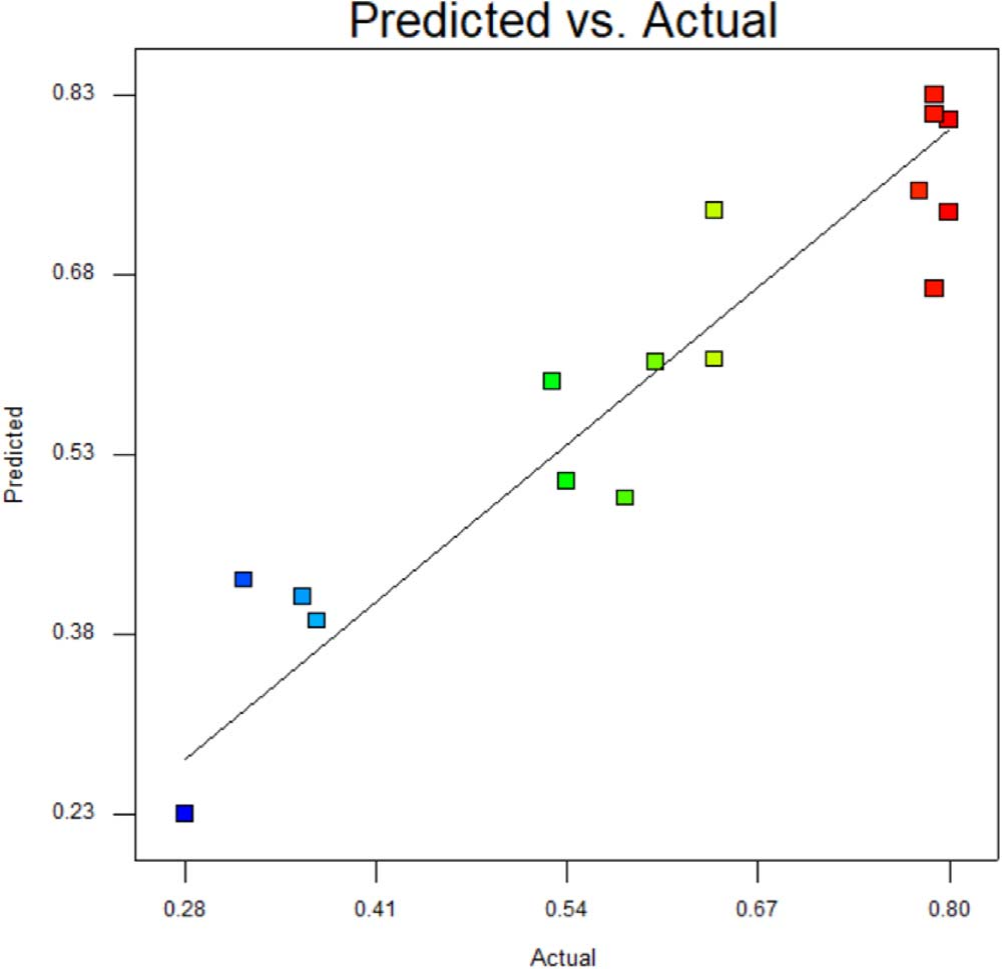
Comparison of experimental data with TLFA predictions.

**Fig. 5:**
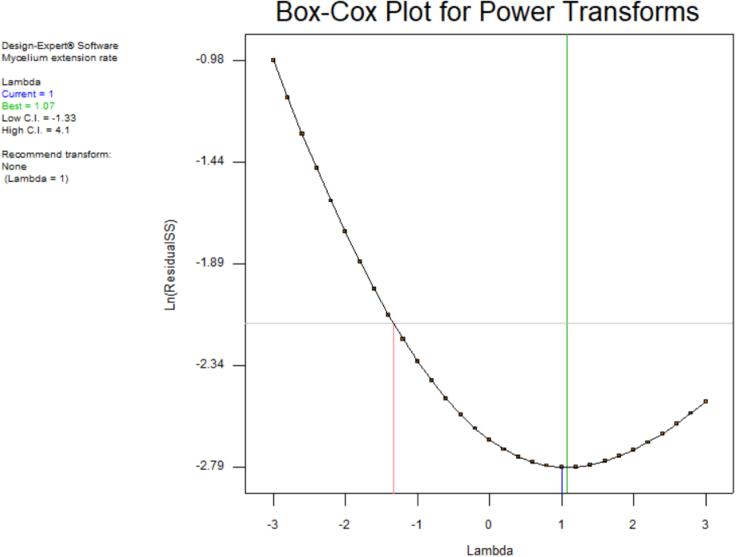
Box-Cox plot of TLFA.

Table 3 shows the percentage contribution for each factor to mycelium extension rate (*M*). Analysis of Variance (ANOVA) (*F*-test) and *p*-value for *M* was used to estimate the coefficients of the model, to check the significance of each parameter, and to indicate the interaction strength of each parameter. It was observed from the ANOVA analysis that the confidence level was greater than 95% while p-value of the model was less than 0.0313. The model with the *p*-value less than 0.05 was statistically significant, which implied that the model was suitable for the experiment. *p*-value for type of substrate (0.3957), size of substrate (0.4726), SP/SS (0.2895), temperature (0.2895) and pretreatment of substrate (0.0015). In this case, pretreatment of substrate is the most significant since it has p-value less than 0.05. This also was supported by the information in Pareto Chart (Fig. 1). The coefficient of determination (*R*^2^) of this model was 0.8829. In details, Fig. 2 shows the effect of the most contributing factors to *M*. The normal probability plot of the residuals and the parity plot comparing the experimental data and predicted have been shown in Figs. 3 and 4 respectively, meanwhile The Box-Cox plot of a natural log (Ln) of the residual sum of square vs lambda has been shown in Fig. 5.

**Table 3:** Factors contribution to mycelium growth extension rate (*M*).

Factor	% Contribution
A - Type of substrate	1.63
B - Size of substrate	1.15
C - SP/SS	2.63
D - Temperature	2.63
E - Pretreatment of substrate	59.30

#### 2.4.2 Nitrogen concentration in mycelium (N)

**Fig. 6:**
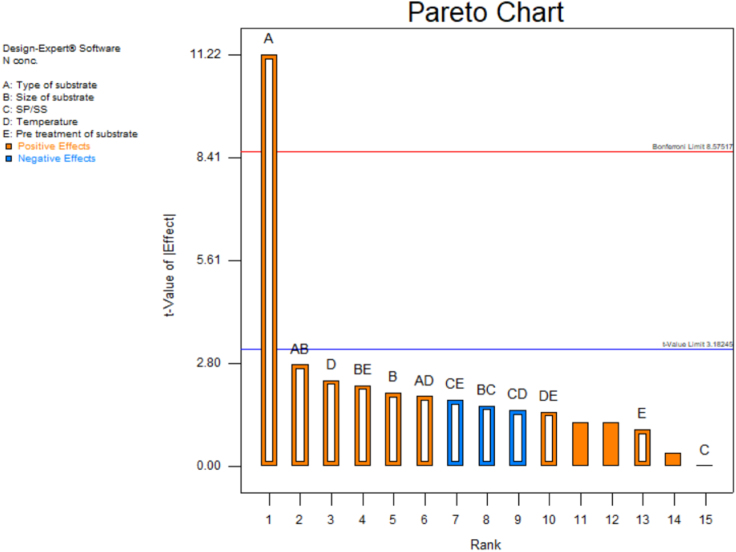
Pareto Chart of nitrogen concentration (*N*).

**Fig. 7:**
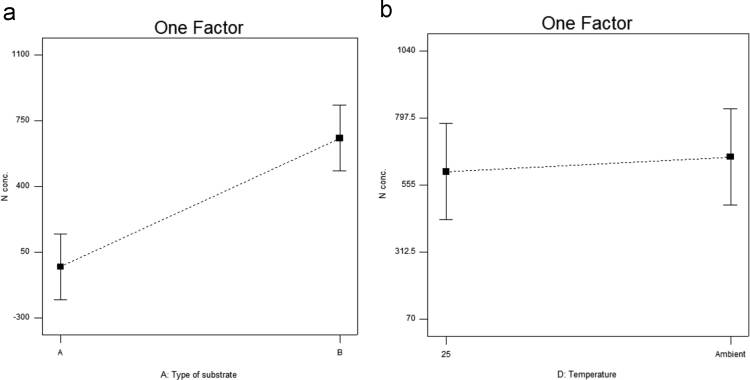
Effect of most contributing factors to nitrogen concentration (*N*).

**Fig. 8:**
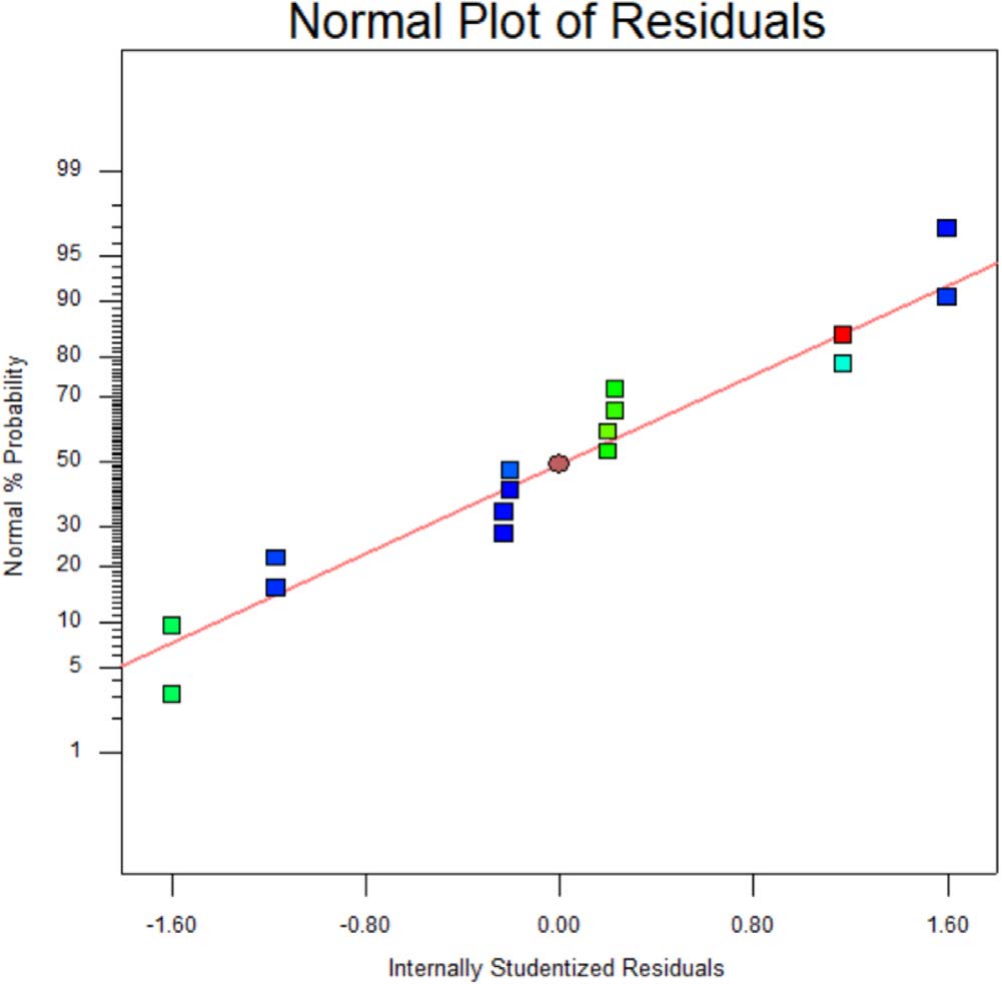
Normal probability plots of internally studentized residuals for nitrogen concentration (*N*).

**Fig. 9:**
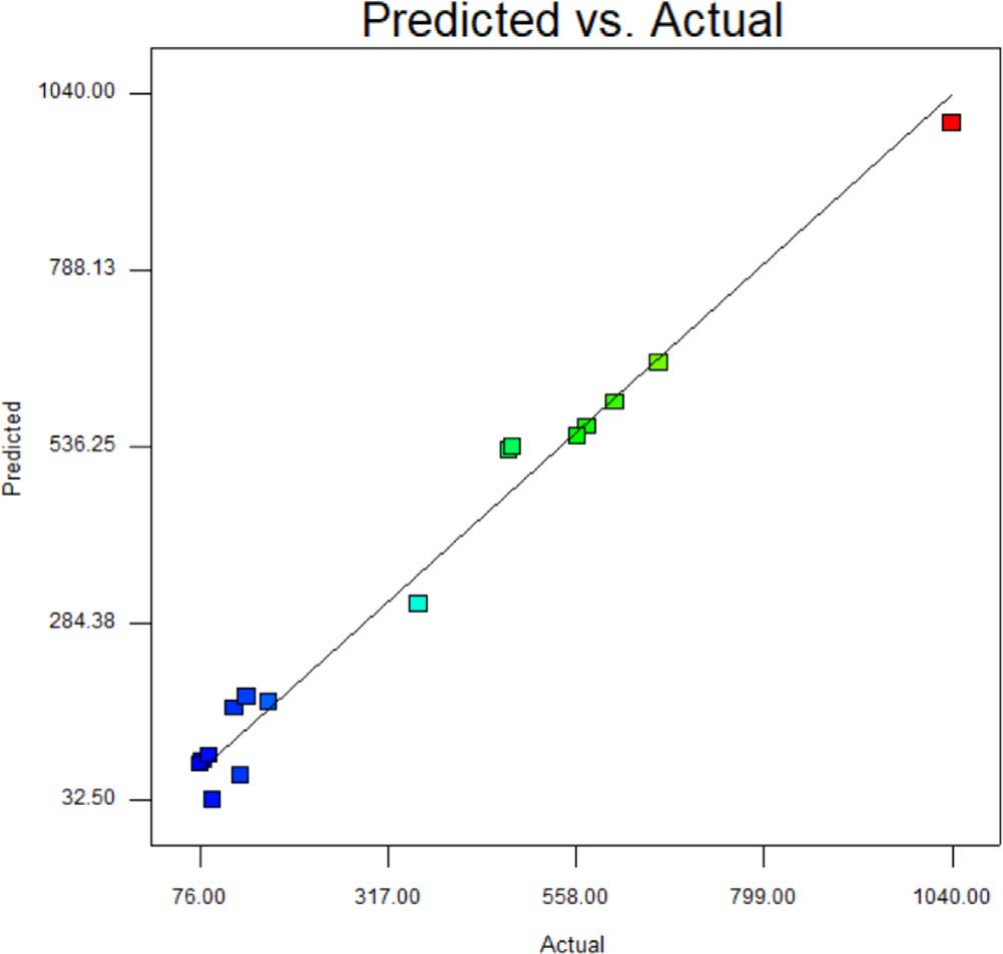
Comparison of experimental data with TLFA predictions.

**Fig. 10:**
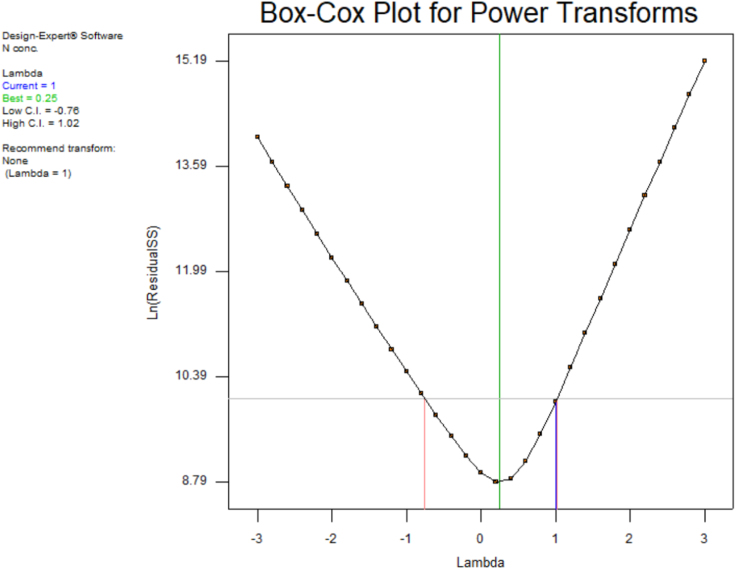
Box-Cox plot of TLFA.

Table 4 shows the percentage contribution for each factor to nitrogen concentration in mycelium (*N*). Analysis of Variance (ANOVA) observed that the confidence level was greater than 95% while *p*-value of the model was less than 0.0313. *p*-value for type of substrate (0.0015), size of substrate (0.1397), SP/SS (0.9829), temperature (0.1007) and pretreatment of substrate (0.3915). In this case, type of substrate is the most significant since it has *p*-value less than 0.05. This also was supported by the information in Pareto Chart (Fig. 6). This model showed *R*^2^ was 0.9819. Fig. 7 shows the effect of the most contributing factors in *M*. The normal probability plot of the residuals and the parity plot comparing the experimental data and predicted have been shown in Figs. 8 and 9 respectively, meanwhile The Box-Cox plot of a natural log (Ln) of the residual sum of square vs lambda has been shown in Fig. 10.

**Table 4:** Factors contribution to nitrogen concentration in mycelium (*N*).

Factor	% Contribution
A - Type of substrate	75.80
B - Size of substrate	2.40
C - SP/SS	0.0003249
D - Temperature	3.31
E - Pretreatment of substrate	0.60

### 2.5 Validation experiment

Validation experiment was conducted based on the suggested best conditions from Design Expert software (Table 5). From the experiment, the maximum values of *M* and *N* were 0.8 cm/day and 656 mg/L respectively.

**Table 5:** Suggested best conditions from Design Expert software.

**Name**	**Suggested value**
Type of substrate	B
Size of substrate	2.5 cm
SP/SS	1:14
Temperature	Ambient
Pretreatment of substrate	Steam
Mycelium extension rate (*M*)	Maximize
Nitrogen concentration (*N*)	Maximize
