# Supplementary material for: Data on modeling mycelium growth in Pleurotus sp. cultivation by using agricultural wastes via two level factorial analysis
Source: Data Brief. 2018 Sep 8;20:1710–20. doi: 10.1016/j.dib.2018.09.008 (PMC6157459; doi:10.1016/j.dib.2018.09.008)

Authors

Noor Athirah Dzulkefli and Assoc. Prof. Ir. Dr. Norazwina Zainol*

Title

Factorial Analysis of Mycelium Growth in Pleurotus sp. Cultivation by using Agricultural Wastes

Abstract

The purpose of this study is to evaluate the factors affecting of mycelium growth in *Pleurotus* sp. cultivation by using agricultural wastes. Two different substrates were used which were empty fruit bunch (*EFB*) and sugarcane bagasse (*SB*). Both substrate were prepared as the selected factors which were type of substrate (*SB* and *EFB*), size of substrates (0.5 cm and 2.5 cm), mass ratio of spawn to substrate (*SP/SS*) (1:10 and 1:14), temperature (25°C and ambient) and pre treatment of substrates (steam and non steam). The responses were mycelium extension rate (*M*) and nitrogen concentration in mycelium (*N*). Design Expert software was used to construct experimental design where all the factor were randomized. As a results, pre treatment and type of substrate is the most contributing factors on both M and N, respectively. It can be concluded that the agricultural waste such as *EFB* can replace sawdust as a media for the *Pleurotus* sp. cultivation.

Keywords: Agricultural wastes; mycelium growth; *Pleurotus* sp.; Two Level Factorial Analysis (*TLFA*)

Author Affiliations

Faculty of Chemical & Natural Resources Engineering, Universiti Malaysia Pahang, 26300 Gambang, Pahang, Malaysia.

*Corresponding author: azwina@ump.edu.my

1. Introduction

Oyster mushrooms are belong to the genus of *Pleurotus* [1]. Mushroom growth has 3 stages which are mycelium growth (spawn running), pinhead formation and fruiting body development

[2]. The growth stage of fungal mycelium in the substrate is very important for mushroom production, since suitable substrate facilitate mycelium colonization and avoid risks of contamination during fruiting body development [3]. It also creates suitable internal conditions for fruiting in order to have high yield of oyster mushrooms [4]. Mushroom requires carbon, nitrogen and inorganic compounds especially at the stage of mycelium growth as its nutritional sources for growth. Nitrogen is an essential element required by all fungi for synthesis of nitrogen for cell component chitin which is composed of ß(1-4)-linked unit N-acetylglucosamine [5]. Neelam *et al*. [6] proved that the ammonium chloride is the major component of nitrogen in the substrate that supported growth of mycelium of *P.florida* and *P.ostreatus*. Ortega *et al*. [7], in their studies with *Pleurotus* sp. described a nitrogen increase in mushroom mycelium related to the amount of nitrogen presented in the initial substrate plus the nitrogen amount in the inoculums.

In the context of Malaysia, even though the oyster mushroom cultivation has a good market potential but the fresh stocks of fresh mushrooms in the market are still inadequate to meet local demands [8]. Rubber sawdust is commonly used in Malaysia as a media for oyster mushroom cultivation. However, the low availability of rubber tree has become serious problem to the mushrooms grower. Thus, the new alternative substrates need to be used to overcome the shortage of mushroom production [9]. Sugarcane waste, paddy straw and tea waste [10] can be used as the new alternative substrate. In order to find the new alternative substrates for *Pleurotus* sp. cultivation, there were a few factors that give contribution in the cultivation such as type of substrate, pH of substrate, moisture content, temperature, size of substrates, mass ratio of spawns to substrates and pre-treatment of substrate [11]. The objective of this study is to evaluate the factors effecting of mycelium growth in *Pleurotus* sp. cultivation by using agricultural wastes. The factors were type of substrate (*SB-A* and *EFB-B*), size of substrates (0.5 cm and 2.5 cm), mass ratio of spawn to substrate (*SP/SS*) (1:10 and 1:14), temperature during spawn running (25°C and ambient) and pre treatment of substrates (steam and non steam). The most contributing factors and interaction between the factors were analyzed via two level factorial analysis (*TLFA*).

2. Materials and Methods

Collection of substrates and spawns

The substrates which were empty palm fruit bunch (*EFB*) were collected from palm oil plantation, while coconut fiber (*CF*) were collected from coconut plantation and banana stem (*BS*) from banana farm at Banting, Selangor. While sugarcane bagasse (*SB*) was collected at Semenyih. Selangor, coffee ground (*CG*) and egg trays (*ET*) were obtained from home.

Preliminary experiment

During first preliminary experiment, the oyster mushroom were cultivated by using five different substrates which were *EFB*, *CF*, *BS*, *ET* and *CG*. The *EFB*, *CF* and *ET* were soaked in water for overnight [12] without cutting into pieces. Meanwhile, *CG* was soaked in water for 30 minutes. Then, the substrates were filtered to drain excess water [4]. The substrates were mixed with oyster mushroom spawn while for *BS*, two holes were made for 1 inch of depth and a few spawns were put in each holes [13]. All substrates were leaved in the dark condition at ambient (28 to 30°C) temperature until the substrates were fully colonized with mycelium.

During second preliminary experiment, *ET* was changed to *SB* substrate. Hundred gram of *EFB*, *CF*, *SB* and *BS* were cut into 2.5 cm and soaked in water for overnight while *CG* was soaked in water for 15 minutes and drained the excess water [14]. The substrates were mixed with oyster mushroom spawn and leaved in the dark condition at ambient temperature until the substrates were fully colonized with mycelium.

During third preliminary experiment, only *SB* substrate was prepared and inoculated with oyster mushroom spawn in order to measure the mycelium growth extension (*M*). The *SB* was prepared as second preliminary experiment and the spawn was placed on the surface of substrate so that the mycelium spreading from the surface towards the bottom of the container [4].

Therefore, from the preliminary experiment, two substrates were chosen for factorial analysis experiment based on the highest mycelium extension rate (*M*).

Experimental set-up for factorial analysis

There were five selected factors that give contribution to oyster mushroom growth (Tab. 1). The factors were type of substrates, size of substrates, mass ratio of spawn to substrates (*SP/SS*), temperature and pre-treatment of substrates. *SB* (*A*) and *EFB* (*B*) were prepared in bottles according to run in Tab. 2. Firstly, the substrates were cut into the selected size, then soaked in water for overnight, filtered to drain excess water and weighed for 100 g. Then, the substrates were pre treated with selected pre treatment and inoculated with spawn by placing the spawn on the surface of substrate. The bottles were closed and incubated at selected temperature in the dark condition. The experiment set-up in Tab. 2 was performed by Design Expert software where all the factors were randomized [15]. Then, experimental data were analyzed by using Design Expert software in order to determine the most contributing factors. The experiment was conducted according to set-up in Tab. 2. There were two responses which were mycelium extension rate (*M*) and nitrogen concentration in mycelium (*N*).

Table 1

Table 2

Validation experiment

Validation experiment was conducted based on the suggested best conditions from Design Expert software. The criteria setup to select the best processing conditions were given in Tab. 3. All factors were set as in range. Meanwhile, the responses were set to get the maximum value.

Table 3

Sample analysis

Sample analysis was conducted after all run fully colonized with mycelium. There were two responses which were mycelium extension rate (*M*) and nitrogen concentration in mycelium (*N*).

Determination of mycelium growth

Spawn run (mycelium extension) was observed regularly until appears white colonization. Each experiment was determined the days for complete mycelium growth. Mycelium growth was

measured in centimeters as the length of the mycelium spreading from the surface of substrates toward the bottom of bottles [4].

Nitrogen concentration analysis by using HACH Spectrophotometer

N was determined by using Persulfate Digestion Method (Method 10072). The mycelium was collected from the substrates and diluted by using deionized water using 40 dilution factor. Then, *N* was analyzed by using HACH Spectrophotometer.

Data analysis

All data obtained were recorded in Design Expert software. The responses were analyzed using Analysis of Variance (*ANOVA)* based on p-value with 95% of confidence level to identify the most contributing factors and interaction between the factors that has an effect on both responses *M* and *N*.

3. Results and Discussions

Preliminary experiments

From Fig. 1, the mycelium growth in the substrates were observed. After 12 days, *EFB* and *CF* showed high amount of mycelium but other substrates showed less mycelium growth in the substrates. This may be due to ability of *EFB* and *CF* retain moisture content for long period of time [16]. According to Chang and Miles [17], appropriate moisture in the substrate should encompass a range between 50% and 75% in the substrate, enabling the satisfactory growth of *Pleurotus* sp. mycelium. However, both seemed to have longer time to fully colonized because the substrates were not cut into smaller size, so the surface area of the substrates were lower. Substrates need to be cut into 2-6 cm to provide a larger surface area, thus improving the oxygen transfer to the substrate [18]. Besides, the mycelium in both *BS* and *ET* showed keep increasing in the early days but started to decrease after 9 days. This may be due to the loss of moisture content of the substrates and inappropriate mass ratio of spawn to substrates. Therefore, the spawn could not obtained enough nutrients from the substrates because of low moisture content in the substrate will result in difficult breathing for the mycelium [11].

Figure 1

For second preliminary study, *SB* was used instead of *ET* because *SB* has higher nitrogen content [9] and can retain moisture content longer [19]. Nitrogen is the main nutrient for mycelium growth. The high nitrogen content will result the shorter time to complete mycelium growth [20]. The mass of substrate was weighed to determine the performance of mycelium growth. The mass of substrates showed no changes after 10 days. Besides, the spawn did not showed any changes and no mycelium was produced. This may be due to the ineffective oyster mushroom spawn because the spawn was not stored at lower temperature.

During third preliminary study, only sugarcane bagasse substrate was used and inoculated with oyster mushroom spawn in order to measure the mycelium growth extension. The method from second preliminary experiment to measure the performance of mycelium growth was not suitable to be used. Therefore, the mycelium growth was measured as the mycelium spreading from the surface of substrate to the bottom. The mycelium extension in the *SB* is 0.28 cm/day. The study conducted by Hoa *et al*. [9] showed that, the *SB* has higher nitrogen (*N*), carbon (*C*) and *C/N* ratio which were 1.20%, 55.00% and 45.83, respectively compared to sawdust. Naraian *et al*. [21] reported that the mycelium growth and primodial development of *Pleurotus florida* were dependent on the lignocellulosic materials, especially *C/N* ratio. Yang [22] also reported that higher *C/N* ratio favored the mycelium growth and lower *C/N* ratio favored fruiting body growth.

In sum, from first preliminary experiment, *EFB* has the highest amount of mycelium. The experiment was stopped after 12 days since all substrate took longer time to fully colonize. During second preliminary experiment, the experiment was unsuccessful because the oyster mushroom spawn used was ineffective, therefore no mycelium was produced after 10 days. Besides, the method to measure the mycelium growth in the substrates was not suitable. During third preliminary experiment, the correct method to measure the mycelium growth and new oyster mushroom spawn was used. The result *SB* recorded 0.28 cm/day *M*. It showed positive growth from *SB*. Therefore, *EFB* and *SB* was used for factorial analysis.

Factorial analysis

Tab. 4 shows 16 runs of experiments were done for this study and the results of mycelium extension rate (*M*) and nitrogen concentration (*N*). The responses were analyzed using *ANOVA* by using Design Expert software V7, based on the value with 95% confidence level to identify

the most contributing factors and interaction between the factors on both responses *M* and *N*. *M* was ranged from 0.28 to 0.80 cm/day while *N* was ranged from 76 to 1040 mg/L. The lowest value of 0.28 cm/day of *M* was obtained at substrate *B*, 2.5 cm size of substrate, 1:14 *SP/SS*, incubated at ambient temperature and application of steam treatment; the highest value of 0.80 cm/day of *M* was obtained at substrate *B*, 2.5 cm size of substrate, 1:10 and 1:14 *SP/SS*, incubated at ambient temperature and no application of steam treatment. Meanwhile, the lowest value of 76 mg/L of *N* was obtained at substrate *A*, 2.5 cm size of substrate, 1:14 *SP/SS*, incubated at ambient temperature no application of steam treatment; the highest value of 1040 mg/L of *N* was obtained at substrate *B*, 2.5 cm size of substrate, 1:10 *SP/SS*, 25°C and application of steam treatment.

Table 4

Analysis of variance (*ANOVA*) for mycelium extension rate (*M*)

Tab. 5 shows the percentage contribution for each factor towards mycelium extension rate (*M*) which is pre treatment of substrate (*E*) has the highest percentage with value of 59.30%, followed by *SP/SS* (*C*) and temperature (*D*), type of substrate (*A*) and lastly size of substrate (*B*) has the least percentage with value 1.15%. *ANOVA* summary was shown in Tab. 6 for mycelium extension rate (*M*) to estimate the coefficient of the model, check the significance of each parameter and indicate the interaction strength of each parameter. This model showed the coefficient of determination (R^2) was 0.8829. Olmez [23] suggest that a good fit of a bioprocess model, R^2 should be at least 0.80. Since the R^2 for this response variables is higher than 0.8, this model was accepted. It can be concluded that this model can be used for optimization.

Table 5

Table 6

Main effect and interaction effect between factors on mycelium extension rate (*M*)

The Pareto Chart in Fig. 2 shows the main effects and interaction effects of the factors for *M*. For the main effect, it showed that there are three main factors contribute to *M*. Pre treatment of

substrate (*E*) has shown the highest effects followed by *SP/SS* (*C*) and temperature (*D*). In Tab. 5, Factor *E* does give the highest contribution among other factors. Based on Tab. 1, for Factor *E*, steam was set as low level and non steam was set as high level, meanwhile Factor *C*, 1:10 was set as low level and 1:14 was set as high level. Negative effect is when the factor is not proportional to the response value. From Fig. 2, Factor *E* and Factor *C* gave negative effect towards the *M*. Therefore, when the Factor *E* and Factor C is at low level, the value of *M* is increasing. On the other hand, Factor *D* gave positive effect towards the *M*. Positive effects is when the factor is proportional to the response value. Therefore, when Factor *D* is increasing, the value of *M* is increasing as well. For interaction effects, it showed there were two interaction effects that contributed in *M* which were *SP/SS* and pre treatment of substrate (*CE*) and temperature and pre treatment of substrate (*DE*) with negative and positive effects respectively.

Figure 2

Effect of independent processing parameters on mycelium extension rate (*M*)

The effect of three independent variables on *M* which were *SP/SS*, temperature and pre treatment of substrate are shown in Fig. 3a, Fig. 3b and Fig. 3c respectively. From Fig. 3a, there was no significant difference of *SP/SS* on *M* because at ratio 1:14, *M* achieved 0.81 cm/day while at ratio 1:10 achieved 0.78 cm/day. This is supported by the information from Pareto Chart (Fig. 2) where *SP/SS* factor is below than t-value limit. Banala *et al*. [24] reported that coefficients with t-value of effect above Bonferroni line are designated as certainly significant coefficients, and coefficients with t-value of the effect below the t-limit line is statistially insignificant to the response. From Fig. 3b, at ambient (28 to 30°C) temperature, *M* achieved 0.81 cm/day but at 25°C achieved 0.73 cm/day which was lower than ambient temperature. It showed there was no significant difference between both temperatures. This is because the range of temperature used in this study was in the range of optimum temperature (25 to 30°C) for mycelium growth of oyster mushroom *Pleurotus* spp. [6]. This finding similar with the studies of Hoa and Wang [25] wheras the obtained optimum temperature for *Pleurotus* species was found to be in the range of 24°C to 32°C (ambient). From Fig. 3c, *M* is at 0.61 cm/day when there was no treatment applied and the value of *M* become higher which is 0.81 cm/day when steam treatment was applied. This is because steam pasteurization can reduce the amount of microscopic competitors in substrates.

This gives the mycelium an advantage over harmful organisms, allowing it to take over the substrate and eventually produce mushrooms [26]. Results obtained contradicts with Oseni *et al*. [27] who obtained full colonization of *Pleurotus* sp. with 0.41 cm/day on sugarcane bagasse.

Figure 3

Interaction effects between factors on mycelium extension rate (*M*)

Fig. 4a shows the interaction effect between *SP/SS* and pre treatment of substrate (*CE*) on *M*. When steam treatment (red line) was applied, it gives better performance on *M*. There was no significant difference of *SP/SS* on *M* because at ratio 1:14, *M* achieved 0.81 cm/day while at ratio 1:10 *M* achieved 0.78 cm/day. Meanwhile, when no treatment (green line) was applied, it showed significant difference of *SP/SS* on *M*. As indicated by Pareto Chart (Fig. 2), the most significant factor is pre treatment because it is above Bonferroni Limit while *SP/SS* is insignificant factor to *M* since it is below than t-limit. Therefore, *M* showed significant difference when no treatment was applied. At ratio 1:10, *M* achieved 0.60 cm/day and the value become lower which is 0.46 cm/day at ratio 1:14. According to Beyer [28], by pasteurizing the substrate with steam can optimize the condition of the substrate by reducing or eliminating the bad microorganism and prevent competition between the spawn and other microorganism. When there was no application of steam to substrate, spawn needs to compete for the substrate with other microorganism. Thus, more spawn will gives better performance of *M* [29]. In this study, the amount of spawn at ratio 1:10 is more than at ratio 1:14, therefore *M* achieved better performance at ratio 1:10. Fig. 4b shows the interaction effect between temperature and pre treatment of substrate (*DE*) on *M*. It was the same as interaction effect of *CE* where *M* recorded higher when steam (red line) treatment was applied. When steam treatment was applied, there was no significant difference of temperature on *M* because *M* achieved 0.81 cm/day at ambient (28 to 30°C) temperature and 0.78 cm/day at 25°C. Meanwhile, when no treatment (green line) was applied, *M* is higher at ambient temperature because *Pleurotus* sp. best grow at 28 to 30°C [25].

Figure 4

Analysis of variance (*ANOVA*) for nitrogen concentration in mycelium (*N*)

Tab. 7 shows the percentage contribution for each factor towards nitrogen concentration in mycelium (*N*) which is type of substrate (*A*) has the highest percentage with value of 75.80%, followed temperature (*D*), size of substrate (*B*), pre treatment of substrate (*E*) and lastly *SP/SS* has the least percentage with value 0.0003%. *ANOVA* summary was shown in Tab. 8. This model showed R^2 was 0.8829. Since the R^2 for this response variables is higher than 0.8, this model was accepted. It can be concluded that this model can be used for optimization.

Table 7

Table 8

Main effect and interaction effect between factors on nitrogen concentration in mycelium (*N*)

The Pareto Chart in Fig. 5 shows the main effects and interaction effects of the factors for *N*. For the main effect, it showed that there is only one main factors contribute to *N*. Type of substrate (*A*) has shown the highest effects. In Tab. 7, Factor *A* does give the highest contribution among other factors. Based on Tab. 1, for Factor *A*, *SB* was set as low level and *EFB* was set as high level. Positive effect is when the factor is proportional to the response value. From Fig. 4, Factor *A* gave positive effect towards the *N*. Therefore, when the Factor *A* is at high level, the value of *N* is increasing. For interaction effects, it showed there was one interaction effects that contributed in *M* which was type of substrate and size of substrate (*AB*) with positive effect as well.

Figure 5

Effect of independent processing parameters on nitrogen concentration in mycelium (*N*)

The effect of two independent variables on *N* which were type of substrate and temperature is shown in Fig. 6. Type of substrate shows an important effect on *N* value (Fig. 6a). As supported by Pareto Chart (Fig. 5), the type of substrate is the most significant factor to *N* because it is above Bonferroni Limit. It showed that *N* achieved 656 mg/L when substrate *B* (*EFB*) was used. However, the value of *N* become lower (27.5 mg/L) when substrate *A* (*SB*) was used. According

to Widiastuti and Tri-Panji [30] and Hoa *et al*. [9], nitrogen content in *EFB* and *SB* was 2.34% and 1.20%, respectively. Ortega *et al*. [7] described a nitrogen increase in mushroom mycelium related to the amount of nitrogen in the initial substrate. Therefore, the higher the *N* content in substrate, the higher the nitrogen concentration in mycelium. Therefore, the higher the *N* content in substrate, the higher the nitrogen concentration in mycelium. Fig. 6b shows the *N* was 656 mg/L at ambient temperature while it become lower (603 mg/L) at 25°C. According to Cheng *et al*. [31], the main nitrogen sources to get high dry weight of mycelium were the ammonium chloride and ammonium sulfate and it work best in the range of 28°C to 30°C [6] which in this study, the ambient temperature was in the range.

Figure 6

Interaction effects between factors on nitrogen concentration in mycelium (*N*)

Fig. 7 shows the interaction effect between the type of substrate and size of substrate (*AB*) on *N*. When using substrate *B*, for both 2.5 cm (red line) and 0.5 cm (black line) size of substrate, the *N* value achieved 664 mg/L and 617 mg/L, respectively which was higher than substrate *A*. Besides, there was no significant difference of size of substrate on *N* when using substrate *B* but it showed the significant difference when using substrate *A*. As indicated by Pareto Chart (Fig. 4), the most significant factor is type of substrate because it is above Bonferroni Limit while size of substrate is insignificant factor to *N* since it is below than t-limit. Therefore, the type of substrate has important effect on *N* value. *N* achieved 171 mg/L at 0.5 cm and become lower which was 27 mg/L at 2.5 cm size of substrate. According to Bellettini *et al*. [11], the substrate that has low nitrogen content in the range of 0.03% to 1.5% can be cut into small size to increase the surface area of the substrate and nitrogen content in the substrate. The substrate *A* (*SB*) has 1.20% nitrogen while substrate *B* (*EFB*) has 2.34% nitrogen, so substrate *A* has lower nitrogen content than substrate *B* [29, 20], thus by cutting into 0.5 cm, it gave better performance on *N* than 2.5 cm.

Figure 7

Validation of experiment

Validation experiment was conducted based on the suggested the best conditions by Design Expert Software (Tab. 9). Three runs were conducted in order to compare the predicted result from Design Expert software and the actual result. The error was calculated by using Equation 1. Tab. 10 shows the comparison of predicted and actual data of M and N. Run 1 was selected due to the lowest value of error compared to others. Thus, the actual results from suggested best condition are 0.8 cm/day of *M* and 656 mg/L of *N*.

$$\text{Error calculation} = \left|\frac{\text{actual} - \text{predict}}{\text{actual}}\right| \times 100 \qquad (1)$$

Table 9

Table 10

4. Conclusion

The purpose of this study is to evaluate the factors affecting the mycelium growth in *Pleurotus* sp. cultivation by using agricultural waste. The results showed that the most contributing factor for mycelium extension rate (*M*) was pre treatment of substrate while the type of substrate was the significant factor for nitrogen concentration (*N*). The best conditions for the mycelium growth of *Pleurotus* sp. cultivation were using substrate *B* (*EFB*), 2.5 cm size of substrate, 1:14 *SP/SS*, incubated at ambient temperature and application of steam treatment to substrate with 0.8 cm/day of *M* and 656 mg/L of *N*. It can be concluded that the agricultural waste such as empty palm fruit bunch (*EFB*) can replace the sawdust as a media for oyster mushroom cultivation.

Acknowledgement

The author wish to acknowledge the Universiti Malaysia Pahang for funding the project under grant RDU170346.

Abbreviations

EFB	Empty palm fruit bunch
SB	Sugarcane bagasse
SP/SS	Mass ratio of spawn to substrate

M	Mycelium extension rate
N	Nitrogen concentration in mycelium
TLFA	Two level factorial analysis
BS	Banana stem
CF	Coconut fiber
CG	Coffee ground
ET	Egg trays
ANOVA	Analysis of variance
A	Type of substrate
B	Size of substrate
C	SP/SS
D	Temperature
E	Pre treatment of substrate

References

[1] W. S. Kong, *Mushroom Growers' Handbook*, 1, MushWorld, 2004

[2] L. Pathmashini, V. Arulnandhy and R. W. Wijeratnam, *Ceylon Journal of Science (Biological Sciences)* 2008,*37 (2)*, DOI: 10.1016/j.jksus.2018.04.021

[3] E. Bernanrdi, E. Minotto and J. S. D. Nascimento, *Arquivos do Instituto Biologico* 2013, *80 (3)*, DOI: 10.1590/S1808-16572013000300009

[4] C. P. Pokhtrel, N. Kalyan and U. Budathoki, *International Journal of Agricultural Policy and Research* 2013 , *1(2)*, DOI: 10.1016/0269-7483(87)90110-8

[5] P. G. Miles, *Mushroom Biology: Concise Basics And Current Developments*, 1, World Scientific, 1997

[6] S. Neelam, S. Chennupati and S. Singh, *Asian Journal of Plant Science and Research* 2013, *3(1)*, www.pelagiaresearchlibrary.com

[7] M. Ortega, E. Martinez and D. Betancourt, *World Journal of Microbiology Biotechnology* 1992., *8(4)*, DOI: 10.1007/BF01198754

[8] https://www.pressreader.com/malaysia/the-star-malaysia/20170221/281621010100603

(Accessed December 7, 2017)

[9] H. T. Hoa, C.-L. Wang and C. H. Wang, *Mycobiology* 2015, *43(4)*, DOI: 10.5941/MYCO.2015.43.4.423

[10] V. Barshteyn and T. Krupodorova, *Journal of microbiology, biotechnology and food sciences* 2016, *5(6)*, DOI: 10.15414/jmbfs.2016.5.6.563-577

[11] M. B. Bellettini, F. A. Fiorda and H. A. Maieves, *Saudi Journal of Biological Sciences* 2016, DOI: 10.1016/j.sjbs.2016.12.005

[12] S. Biswas, M. Datta and S. Ngachan, *Mushrooms: A manual for cultivation*, 2, PHI Learning, 2011

[13] http://www.usahawan.com/idea-bisnes/projek-tanaman-cendawan.html/ (Accessed December 5, 2017)

[14] L. Fan and S. Carlos, *Muhsroom Growers Handbook*, 2, MushWorld, 2005.

[15] https://www.statease.com/pubs/whats_new_in_DX9.pdf (Accessed on March 3, 2018)

[16] R. Wahab, S. M. Mohd Dom and M. T. Mustafa, *Journal of Plant Science* 2015*s*, *10 (5)*, DOI: 10.3923/jps.2015.179.190

[17] X. Chang and P. Miles, *Mushrooms: Cultivation, Nutritional Value, Medicinal Effect and Environmental Impact*, 2, CRC Press, 2004.

[18] A. Pandey, C. Soccol and D. Mitchell, *Process Biochemical* 2000, *35 (10)*, DOI: doi.org/10.1016/S0032-9592(00)00152-7

[19] Z. Hamsalu Mosisa, A. Kebede and V. Preetha, *International Journal of Advanced Research* 2015, 3 (2), http://www.journalijar.com/uploads/614_IJAR-4876.pdf

[20] http://eprints.utm.my/id/eprint/8680/ (Accessed on July 18, 2018)

[21] R. Naraian, R. Sahu and S. Kumar, The *Environmentalist* 2009, *29 (1)*, DOI: 10.1007/s10669-008-9174-4

[22] X. Yang, "Cultivation of edible mushroom," *Beijing: China Agricultural Press*, 2000.

[23] T. Olmez, *Journal of Hazardous Materials* 2009, *162 (2-3)*, DOI: 10.1016/j.jhazmat.2008.06.017

[24] V. T. Banala, B. Srinivasan and D. Rajamanickam, *ISRN Pharmaceutics* 2013, DOI: 10.1155/2013/719196

[25] H. T. Hoa and C.-L. Wang, *Myycobiology* 2015, *43 (4)*, DOI: 10.5941/MYCO.2015.43.4.423

[26] http://www.mushroom-appreciation.com/pasteurize.html#sthash.M7taJPX0.dpbs (Accessed on July 7, 2017)

[27] T. O. Oseni, S. O. Dlamini and D. M. Earnshaw, *International Journal of Agriculture & Biology* 2012*, 14 (2),* DOI: 11–373/MFA/2012/14–2–251–255

[28] https://extension.psu.edu/growing-mushrooms-microbial-activity-in-substrate (Accessed on August 18, 2017)

[29] http://urn.kb.se/resolve?urn=urn:nbn:se:slu:epsilon-s-4236 (Accessed on December 21, 2017)

[30] Widiastuti and Tri-Panji, *Biopropal Industry* 2015, *6 (2)*, https://media.neliti.com/media/publications/54525-ID-none.pdf

[31] Z. Cheng, Q. Wu and J. B. Huang, *African Journal of Agriculture Research* 2013, 8 (33), DOI: 10.5897/AJAR11.2350

[32] S. B. Cho, *Oyster Mushroom Cultivation*, 1,MushWorld, 2004

[33] H. Chanakya, *Energy for Sustainable Development* 2015, *27*, DOI: 10.1016/j.esd.2015.04.007

[34] http://www.disknet.com/indiana_biolab/b062.htm (Accessed on March 13, 2018)

[35] R. Zhang, X. Li and J. Fadel, *Bioresource Technology* 2002, 82 (3), DOI: 10.1016/S0960-8524(01)00188-2

[36] M. Bhatti, M. Jiskani and K. Wagan, *Pakistan Journal of Botany* 2007, 39 (7), http://www.pakbs.org/pjbot/PDFs/39(7)/PJB39(7)2685.pdf

[37] M. A. Ali, S. Hussain and R. Nawaz, *Journal of Agricultural Residues* 2004, 42 (2), http://apply.jar.punjab.gov.pk/upload/1374495317_75_Microsoft_Word_-_201-209.pdf

Tables with headings

Table 1: Selected factor and their range

Factors	Low level	High level

Type of substrate	*A*	*B*
Size of substrates	0.5 cm	2.5 cm
SP/SS	1:10	1:14
Temperature during spawn running	25°C	Ambient
Pre-treatment of substrates	Steam	Non steam

Table 2: Experimental setup that has been constructed by using *TLFA* by Design Expert software (Version 7)

	Factor 1	Factor 2	Factor 3	Factor 4	Factor 5
Run	A: Type of substrate	B: Size of substrates (cm)	C: *SP/SS* (g:g)	D: Temperature	E: Pre-treatment of substrate
5	A	0.5	1:10	25 °C	Steam
8	B	0.5	1:10	25 °C	Non-Steam
9	A	2.5	1:10	25 °C	Non-Steam
14	B	2.5	1:10	25 °C	Steam
3	A	0.5	1:14	25 °C	Non-Steam
13	B	0.5	1:14	25 °C	Steam
4	A	2.5	1:14	25 °C	Steam
2	B	2.5	1:14	25 °C	Non-Steam
15	A	0.5	1:10	Ambient	Non-Steam
7	B	0.5	1:10	Ambient	Steam
16	A	2.5	1:10	Ambient	Steam
11	B	2.5	1:10	Ambient	Non-Steam
6	A	0.5	1:14	Ambient	Steam
12	B	0.5	1:14	Ambient	Non-Steam
1	A	2.5	1:14	Ambient	Non-Steam
10	B	2.5	1:14	Ambient	Steam

Table 3: Criteria for validation experiment

Name	Goal	Value
Type of substrate	Is in range	*A* - *B*
Size of substrate	Is in range	0.5 cm - 2.5 cm
SP/SS	Is in range	1:10 - 1:14
Temperature	Is in range	25°C - Ambient
Pre treatment of substrate	Is in range	Steam - Non steam
Mycelium extension rate (M)	Maximize	-
Nitrogen concentration (*N*)	Maximize	-

Table 4: Experimental results of mycelium extension rate (*M*) and nitrogen concentration (*N*)

Run	Factor 1	Factor 2	Factor 3	Factor 4	Factor 5	Response 1	Response 2
	A: Type of substrate	B: Size of substrates (cm)	C: *SP/SS* (g:g)	D: Temperature	E: Pre-treatment of substrate	Mycelium extension rate (*M*) (cm/day)	Nitrogen conc. (*N*) (mg/L)
5	A	0.5	1:10	25°C	Steam	0.60	136
8	B	0.5	1:10	25°C	Non-Steam	0.53	476
9	A	2.5	1:10	25°C	Non-Steam	0.79	92
14	B	2.5	1:10	25°C	Steam	0.64	1040
3	A	0.5	1:14	25°C	Non-Steam	0.79	164
13	B	0.5	1:14	25°C	Steam	0.36	560
4	A	2.5	1:14	25°C	Steam	0.54	88
2	B	2.5	1:14	25°C	Non-Steam	0.79	664
15	A	0.5	1:10	Ambient	Non-Steam	0.78	128
7	B	0.5	1:10	Ambient	Steam	0.58	356
16	A	2.5	1:10	Ambient	Steam	0.32	120
11	B	2.5	1:10	Ambient	Non-Steam	0.80	472
6	A	0.5	1:14	Ambient	Steam	0.37	80

12	B	0.5	1:14	Ambient	Non-Steam	0.64	572
1	A	2.5	1:14	Ambient	Non-Steam	0.80	76
10	B	2.5	1:14	Ambient	Steam	0.28	608

Table 5: Contribution factor of mycelium growth extension rate (*M*)

Factor	% Contribution
A - Type of substrate	1.63
B - Size of substrate	1.15
C - *SP/SS*	2.63
D - Temperature	2.63
E - Pre treatment of substrate	59.30

Table 6: *ANOVA* table for mycelium growth extension rate (*M*)

ANOVA for selected factorial model						
Source	Sum of square	Df	Mean Square	F value	P-value Prob > F	
**Model**	**0.46**	**9**	**0.051**	**5.02**	**0.0313**	**Significant**
A - Type of substrate	8.556E-03	1	8.556E-03	0.84	0.3957	
B - Size of substrate	6.006E-03	1	6.006E-03	0.59	0.4726	
C - *SP/SS*	0.014	1	0.014	1.35	0.2895	
D - Temperature	0.014	1	0.014	1.35	0.2895	
E - Pre treatment of substrate	0.31	1	0.31	30.38	0.0015	
BD	0.026	1	0.026	2.58	0.1593	
BE	0.020	1	0.020	1.98	0.2085	
CE	0.032	1	0.032	3.08	0.1298	
DE	0.032	1	0.032	3.08	0.1298	

Table 7: Contribution factors of nitrogen concentration in mycelium (*N*)

Factor	% Contribution
A - Type of substrate	75.80
B - Size of substrate	2.40
C - *SP/SS*	0.0003249
D - Temperature	3.31
E - Pre treatment of substrate	0.60

Table 8: *ANOVA* table for nitrogen concentration in mycelium (*N*)

***ANOVA* for selected factorial model**						
Source	Sum of square	Df	Mean Square	F value	P-value Prob > F	
**Model**	**1.209E+06**	**12**	**1.007E+05**	**13.59**	**0.0270**	**Significant**
A - Type of substrate	9.332E+05	1	9.332E+05	125.85	0.0015	
B - Size of substrate	29584.00	1	29584.00	3.99	0.1397	
C - *SP/SS*	4.00	1	4.00	2.395E-04	0.9829	
D - Temperature	40804.00	1	40804.00	5.50	0.1007	
E - Pre treatment of substrate	7396.00	1	7396.00	1.00	0.3915	
AB	56644.00	1	56644.00	7.64	0.0699	
AD	26896.00	1	26896.00	3.63	0.1529	
BC	20164.00	1	20164.00	2.72	0.1977	
BE	36100.00	1	36100.00	4.87	0.1145	
CD	17424.00	1	17424.00	2.35	0.2228	
CE	24336.00	1	24336.00	3.28	0.1677	
DE	16384.00	1	16384.00	2.21	0.2339	

Table 9: Suggested best condition for *Pleurotus* sp. cultivation

Type of substrate	*B* (*EFB*)
Size of substrate	2.5 cm

SP/SS	1:14
Temperature	Ambient
Pre treatment of substrate	Steam

Table 10: Results from suggested best condition

	Mycelium extension rate (*M*) (cm/day)			Nitrogen concentration (*N*) (mg/L)		
	Predicted	Actual	Error (%)	Predicted	Actual	Error (%)
Run 1	0.8125	0.8	1.5625	656.5	656	0.076
Run 2	0.8125	0.8	1.5625	656.5	645.2	1.751
Run 3	0.8125	0.8	1.5625	656.5	664	1.130

Figure lagends

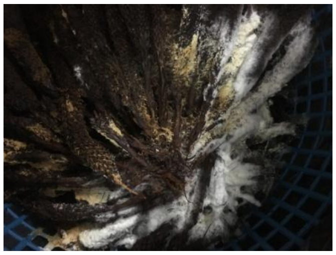

a) Empty Palm Fruit Bunch

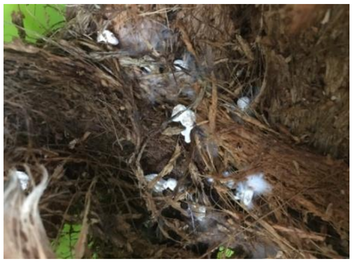

b) Coconut Fiber

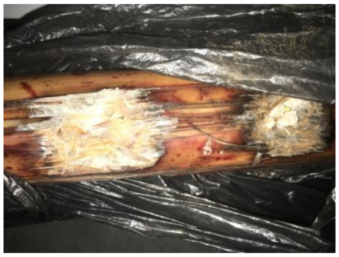

c) Banana stem (*BS*)

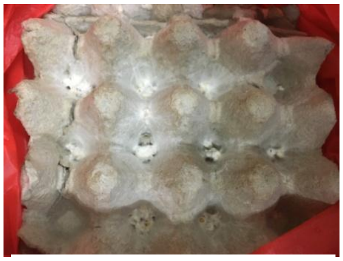

d) Egg Trays (*ET*)

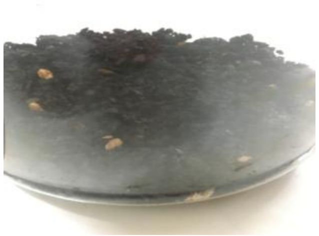

e) Coffee ground (*CG*)

Figure 1: First preliminary experiment after 12 days

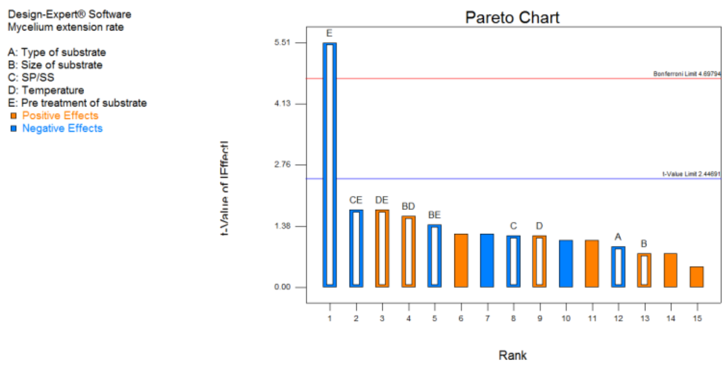

Figure 2: Pareto Chart of mycelium growth extension rate (*M*)

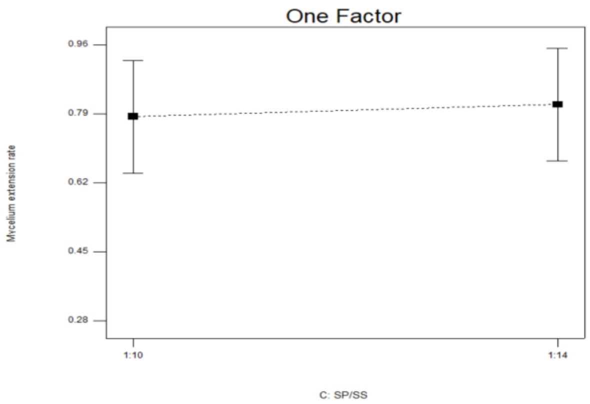

a) *SP/SS*

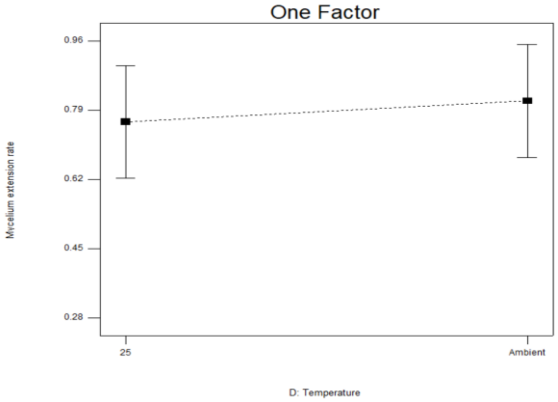

b) Temperature

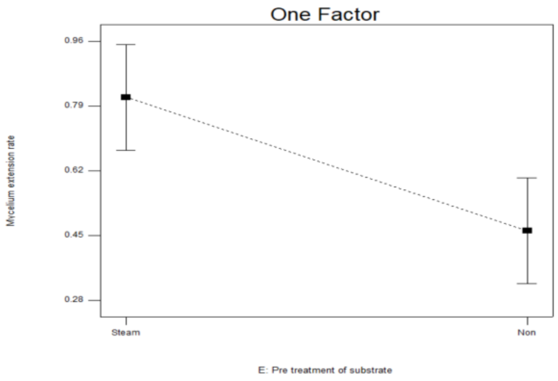

c) Pre treatment of substrates

Figure 3: Effect of most effective independent factors in mycelium extension rate (*M*)

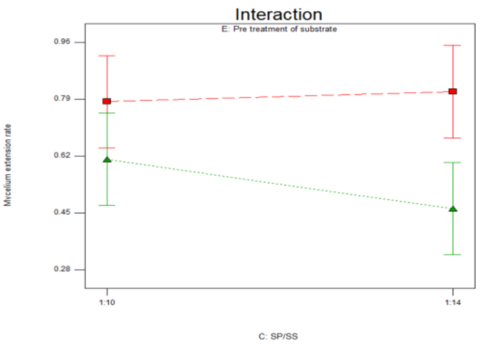

a) Factor SP/SS and pre treatment (*CE*)

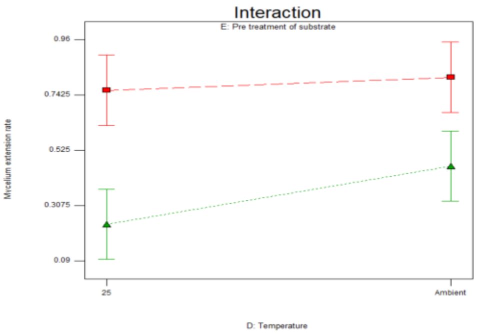

b) Factor of temperature and pre treatment (*DE*)

Figure 4: Analysis of interaction effects on mycelium extension rate (*M*)

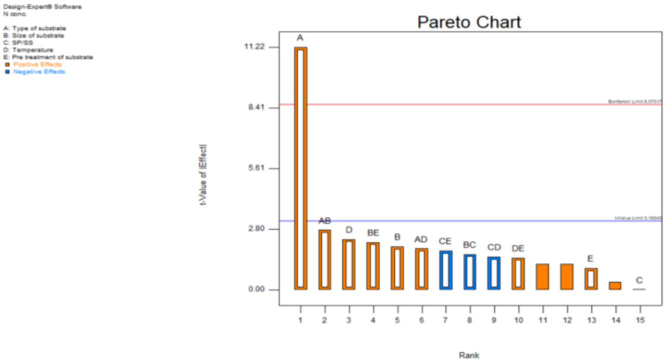

Figure 5: Pareto Chart of nitrogen concentration (*N*)

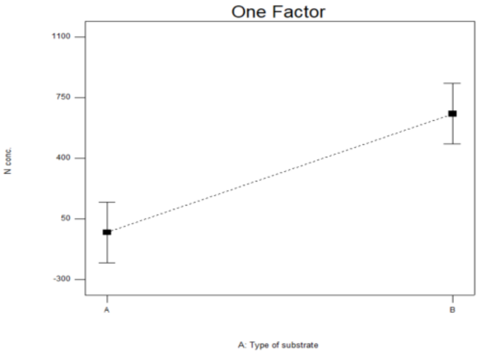

a) Type of substrate

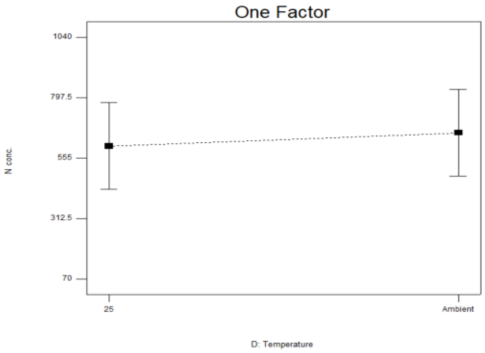

b) Temperature

Figure 6: Analysis of most effective independent factors in nitrogen concentration (*N*)

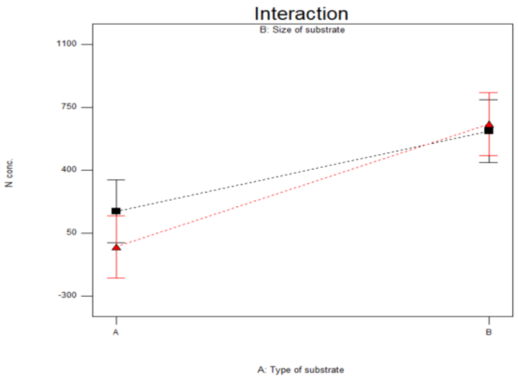

Figure 7: Interaction factors of type of substrate and size of substrate (*AB*)

Table of contents: Graphical Abstract

Oyster mushroom cultivation in Malaysia utilizes sawdust from rubber palm as a substrate for cultivation. However, the low availability of rubber tree has become serious problem for mushroom grower. Empty fruit bunch (*EFB*) was used as a substrate and the results obtained for mycelium rate and nitrogen concentration was 0.8 cm/day and 656 mg/L, respectively. Besides, mushroom grower can cut 60% cultivation cost because the price of EFB is cheaper than sawdust.

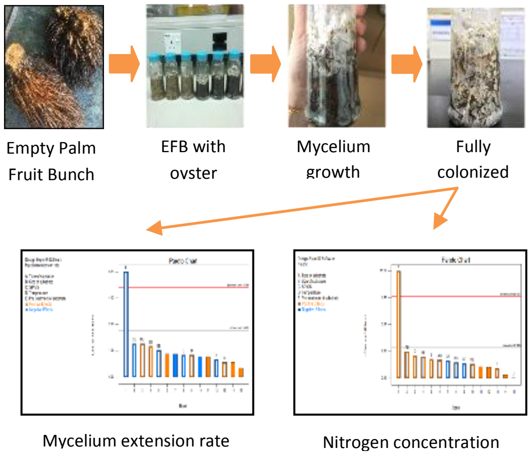

Supplement: Supplementary file 2 — Supplementary material. [file mmc2.pdf]
